# The Impact of Smoking and Obesity on Disability-Free Life Expectancy in Older Australians

**DOI:** 10.1093/gerona/glaa290

**Published:** 2020-11-29

**Authors:** Andrew Kingston, Julie Byles, Kim Kiely, Kaarin J Anstey, Carol Jagger

**Affiliations:** 1 Population Health Sciences Institute, Newcastle University, Newcastle upon Tyne, UK; 2 Research Centre for Generational Health and Ageing, University of Newcastle, New South Wales, Australia; 3 Hunter Medical Research Institute, Newcastle, New South Wales, Australia; 4 School of Psychology, University of New South Wales, Sydney, Australia; 5 Neuroscience Research Australia (NeuRA), Sydney, New South Wales, Australia

**Keywords:** Disablement process, Health expectancy, Obesity, Risk factors

## Abstract

**Background:**

Smoking and obesity are 2 modifiable risk factors for disability. We examine the impact of smoking and obesity on disability-free life expectancy (DFLE) at older ages, using 2 levels of disability.

**Method:**

We used the DYNOPTA dataset, derived by harmonizing and pooling risk factors and disability outcomes from 5 Australian longitudinal aging studies. We defined mobility disability as inability to walk 1 km, and more severe (activities of daily living [ADL]) disability by the inability to dress or bathe. Mortality data for the analytic sample (*N* = 20 401; 81.2% women) were obtained from Government Records via data linkage. We estimated sex-specific total life expectancy, DFLE, and years spent with disability by Interpolated Markov Chain (IMaCh) software for each combination of smoking (never vs ever), obesity (body mass index ≥30 vs 18.5 to <30), and education (left school age 14 or younger vs age 15 or older).

**Results:**

Compared to those without either risk factor, high educated nonobese smokers at age 65 lived shorter lives (men and women: 2.5 years) and fewer years free of mobility disability (men: 2.1 years; women: 2.0 years), with similar results for ADL disability. Obesity had the largest effect on mobility disability in women; high educated obese nonsmoking women lived 1.3 years less than nonsmoking, not obese women but had 5.1 years fewer free of mobility disability and 3.2 fewer free of ADL disability. Differences between risk factor groups were similar for the low educated.

**Conclusions:**

Our findings suggest eliminating obesity would lead to an absolute reduction of disability, particularly in women.

For many countries, life expectancy (LE) is increasing at a faster rate than healthy LE resulting in more years with disability and dependency (1). In England, this is forecast to continue for the next decades, though there is some evidence for compression of dependency in men (2). In Australia, of the years of LE at age 65 gained between 2003 and 2015 (men: 1.9, women: 1.3 years), less than half a year was with disability (men: 0.3, women: 0.1 years) (3). However, although relatively stable, Australian men and women spend 10–12 years with disability at age 65, these constituting over 50% of remaining life. Public health efforts should therefore focus on identifying factors that will delay functional decline to compress the time people spend in receipt of formal care therefore reducing costs to individuals and the state.

Modifiable risk factors, such as obesity and smoking, are obvious intervention targets, although evidence of their impact on the development of functional limitations is equivocal (4–6). Nevertheless, some risk factors for morbidity and disability also increase the risk of death, and so it is vital to assess the impact of these competing risks in a measure such as disability-free life expectancy (DFLE). In this way, we can assess whether prevention of a risk factor will increase DFLE more than LE, thereby adding extra years of independent living and reducing years with disability. Additionally, there is emerging evidence that most of the gain in LE is time spent with milder disability or low levels of dependency rather than at the more severe end of the spectrum (7–9). By understanding the impact of risk factors at milder levels of disability, any interventions deployed to mitigate them could reduce time spent with more severe disability, thereby leading to compression of more acute levels of disability.

One risk factor for disability that has received much attention in recent years is obesity, given its prevalence is increasing worldwide (10,11), and its consequences include increased risk of multimorbidity, disability, frailty, and mortality (12–15). In addition, obesity is associated with conditions that lead to disability. This included falls (16), and musculoskeletal diseases, particularly those that relate to joints that are “stress bearers” (ie, hip and knee) potentially mediated through added strain placed on joints through excess fat (17,18). The incidence of items which measure disability (eg, cutting toenails, using steps, bathing, dressing, feeding) is known to follow a distinct sequence with increasing severity but whether obesity has a differential impact at different stages of this hierarchy is unknown (19). Importantly, the prevalence of obesity for those of low socioeconomic status, those who are female, and those who are older tends to be greater (20,21).

In contrast to obesity, smoking rates have shown declines in a number of countries, including the UK and Australia, although projections suggest that well under half of the countries worldwide will meet the WHO target of reducing adult smoking prevalence by 30% between 2010 and 2025 (22). In particular, smoking remains a public health concern because initiation rates remain high in young adults (23–25). Smoking is a risk factor for many disabling and fatal conditions, including cancers and cardiovascular disease (26). We have previously shown that, compared to nonsmokers, smokers have shorter LE and spend more years with cognitive impairment (27). Additionally, in contrast to obesity, smoking appears to have a much stronger effect on LE than DFLE, though these findings are mostly from European studies (28–31).

In this paper, we examine the impact of obesity and smoking on DFLE at older ages using pooled data from 5 Australian longitudinal studies of aging. In contrast to other studies evaluating the impact of these conditions on DFLE, we investigate 2 measures of disability covering the spectrum of severity.

## Method

The sample was taken from the Dynamic Analyses to Optimise Ageing (DYNOPTA) project in Australia. This dataset consists of pooled data from Australian longitudinal studies of aging which focus specifically on 4 outcomes that contribute to the burden of disease and disability (dementia/cognition, mental health, sensory impairment, mobility/activity limitations) (32). For this analysis, we selected 5 studies that had information on both measures of disability (as well as obesity and smoking): the Australian Longitudinal Study of Ageing (ALSA) covering the period 1992–2000; the Australian Longitudinal Study of Women’s Health (ALSWH) 1996–2008; the Blue Mountains Eye Study (BMES) 1992–2002; the Melbourne Longitudinal Studies on Healthy Ageing (MELSHA) 1994–2004; and the Personality and Health Through Life (PATH) 2001–2005. Full details for each study are published elsewhere (32) but a brief description is provided in Supplementary Methods.

We constructed 2 measures covering the hierarchy of loss of activities of daily living (ADL) (19). The first measure (mobility disability) consisted of 1 item: the ability to walk 1 km with response yes or no. The second measure (ADL disability) included items that examined more severe levels of disability, these being the ability to dress and bathe, and were ascertained by the Short-Form 36 item on dressing or bathing: Does your health now limit you in bathing or dressing yourself? (ALSWH, BMES, PATH), or separate items on difficulty dressing and bathing (ALSA, MELSHA).

Obesity was defined as a body mass index (BMI) of 30 or over; a small number of participants who were underweight (BMI < 18.5) were excluded. Smoking was categorized as current or former smoker versus never smoker. We did not include physical activity as a potential risk factor since this was not sufficiently harmonized across the studies. Education was coded as a binary variable, with those leaving school before age 15 deemed as early school leavers and those who left school aged 15 years or later as late school leavers. Missing values in BMI, smoking, and education were imputed with age, sex, and study using the chained equations method (33). Obesity and smoking status were measured at baseline only.

To assess the impact of obesity and smoking on DFLE through disability and mortality, we used the Interpolated Markov Chain (IMaCh) software (34) version 0.99r19. This technique partitions the time intervals between successive interviews into shorter steps and then models the resulting transition probabilities by multinomial logistic regression on age and any covariates (in our case smoking, obesity, and education). Estimated transition probabilities then act as inputs to a multistate life table. Obesity and smoking were modeled as 2 dummy variables enabling calculation of DFLE for 4 categories: current nonsmokers, not obese; smokers, not obese; nonsmokers, obese; and obese smokers. We performed separate analyses for men and women, and for type of disability: mobility or ADL.

Due to the large size of the ALSWH and its potential impact on results for women, we repeated analyses for women excluding this study.

## Results

The total sample comprised 20 401 people with mean age of 71.3 years (*SD* = 5.7 years). For the combined sample, 81.2% were women but in individual studies, the proportion of women ranged from 48.4% (PATH) to 100% (ALSWH) (Table 1). Levels of education were highest in the 2 studies with the youngest mean age and more recent recruitment (BMES and PATH), and these were the 2 studies with the highest proportion of participants who were obese (Table 1).

**Table 1.** Baseline Characteristics of the Contributing Studies

	ALSA	ALSWH	BMES	MELSHA	PATH
*N*	2087	12 431	2333	1000	2551
Mean age (*SD*)	78.2 (6.7)	72.1 (1.5)	69.6 (8.7)	73.3 (5.9)	62.5 (1.5)
Female, % (*n*)	49.4 (1031)	100 (12 431)	57.5 (1342)	53.3 (533)	48.4 (1234)
Age left school, % (*n*)					
≤14 years	55.3 (1155)	42.5 (5283)	27.1 (633)	50.2 (502)	11.8 (301)
>14 years	43.4 (906)	54.1 (6728)	59.9 (1398)	49.8 (498)	88.0 (2245)
Missing	1.3 (26)	3.4 (420)	12.9 (302)	0.0 (0)	0.2 (4)
Smoking, % (*n*)					
Current or ex	50.3 (1050)	34.8 (4331)	47.3 (1104)	54.5 (545)	48.1 (1227)
Never	48.8 (1018)	58.3 (7243)	49.9 (1165)	44.2 (442)	51.8 (1320)
Missing	0.9 (19)	6.9 (857)	2.7 (64)	1.3 (13)	0.1 (3)
Body mass index, % (*n*)					
Underweight (<18.5)	1.4 (29)	2.9 (360)	1.5 (34)	2.0 (20)	0.8 (20)
Normal (18.5–25)	30.4 (635)	44.8 (5575)	39.7 (927)	35.8 (358)	35.0 (893)
Overweight (25–30)	32.3 (675)	29.8 (3699)	40.6 (948)	39.3 (393)	37.4 (953)
Obese (>30)	10.8 (225)	11.9 (1484)	17.0 (397)	15.5 (155)	17.8 (453)
Missing	25.1 (523)	10.6 (1313)	1.2 (27)	7.4 (74)	9.1 (231)
Mobility disability^a^					
Yes	22.9 (477)	24.9 (3093)	19.6 (456)	8.6 (86)	8.3 (211)
No	76.2 (1591)	68.4 (8499)	69.2 (1614)	91.1 (911)	91.5 (2334)
Missing	0.9 (19)	6.8 (839)	11.3 (263)	0.3 (3)	0.2 (5)
ADL disability^b^					
Yes	10.7 (223)	12.0 (1497)	11.7 (273)	1.3 (13)	4.9 (126)
No	89.1 (1859)	82.3 (10 235)	78.5 (1831)	97.6 (976)	94.9 (2421)
Missing	0.2 (5)	5.6 (699)	9.8 (229)	1.1 (11)	0.1 (3)

*Notes*: ALSA = Australian Longitudinal Study of Ageing; ALSWH = Australian Longitudinal Study of Women’s Health; BMES = Blue Mountains Eye Study; MELSHA = Melbourne Longitudinal Studies on Healthy Ageing; PATH = Personality and Total Health Through Life Study.

^a^Difficulty walking 1 km. ^b^Health now limits bathing or dressing (ALSWH, BMES, PATH), separate items on difficulty dressing and difficulty bathing (ALSA, MELSHA).

Separate models were fitted for men and women and for mobility disability and ADL disability after removal of those participants with missing baseline disability status (*n* = 1129 for mobility disability and *n* = 947 for ADL disability). The number of transitions between disability states and to death by gender are shown in Supplementary Table 1. Men with low education (left school age 14 or younger) were 25% more likely and women with low education were 14% more likely to become mobility disabled compared to their more educated counterparts (relative probabilities and 95% CI: men = 1.25 [1.04, 1.51]; women= 1.14 [1.07, 1.22]). Similar associations were observed for incident ADL disability (Supplementary Table 2). The effect of smoking was evident mostly in the higher probabilities of death with no disability for both men and women, and of death following disability in women only. Obesity on the other hand had a stronger association in women, increasing the likelihood of incident mobility and ADL disability, and reducing the likelihood of recovery from both types of disability. In men, obesity reduced the likelihood of regaining mobility independence.

To investigate the impact of smoking, obesity, or their co-occurrence, we calculated the difference in total years of life, years free of disability, and years with disability at age 65 between having one or more risk factors compared to being a nonsmoker and not obese (normal or overweight BMI range) (mobility disability: Table 2; ADL disability: Table 3). For both types of disability, differences were very similar by level of education; we therefore discuss results for those with high education. Results for the group with low education are provided in Tables 2 and 3.

**Table 2.** Total Life Expectancy, Years Free of Mobility Disability, and Years With Mobility Disability (*SE*s in parentheses) at Age 65, by Risk Factor, Education and Gender

	Total Life Years (*SE*)	Years Free of Mobility Disability (*SE*)	Years With Mobility Disability (*SE*)
Men			
High education (≥14 years)			
Nonsmokers, not obese	20.9 (0.5)	17.0 (0.5)	3.8 (0.3)
Smokers, not obese	18.3 (0.3)	15.0 (0.3)	3.4 (0.2)
Nonsmokers, obese	19.6 (0.7)	15.3 (0.8)	4.3 (0.5)
Smokers, obese	17.5 (0.6)	13.6 (0.6)	3.9 (0.4)
Loss(−)/gain(+)^a^			
Smoker, not obese	−2.5*** (0.6)	−2.1*** (0.6)	−0.5 (0.4)
Nonsmoker obese	−1.3 (0.8)	−1.8 (0.9)	0.5 (0.6)
Smoker, obese	−3.3*** (0.7)	−3.4*** (0.8)	0.1 (0.5)
Low education (<14 years)			
Nonsmokers, not obese	19.9 (0.5)	16.1 (0.5)	3.8 (0.3)
Smokers, not obese	17.5 (0.4)	14.1 (0.4)	3.3 (0.2)
Nonsmokers, obese	18.6 (0.7)	14.3 (0.8)	4.3 (0.5)
Smokers, obese	16.6 (0.6)	12.7 (0.6)	3.9 (0.4)
Loss(−)/gain(+)^a^			
Smoker, not obese	−2.5*** (0.6)	−2.0** (0.6)	−0.5 (0.4)
Nonsmoker obese	−1.3 (0.8)	−1.8* (0.9)	0.5 (0.6)
Smoker, obese	−3.3*** (0.7)	−3.4*** (0.8)	0.1 (0.5)
Women			
High education (≥14 years)			
Nonsmokers, not obese	24.5 (0.2)	16.7 (0.2)	7.9 (0.2)
Smokers, not obese	22.1 (0.2)	14.7 (0.2)	7.4 (0.2)
Nonsmokers, obese	22.9 (0.3)	11.6 (0.3)	11.3 (0.4)
Smokers, obese	20.7 (0.3)	9.9 (0.3)	10.8 (0.4)
Loss(−/gain(+)^a^			
Smoker, not obese	−2.5*** (0.3)	−2.0*** (0.3)	−0.4 (0.3)
Nonsmoker obese	−1.6*** (0.4)	−5.1*** (0.3)	3.5*** (0.4)
Smoker, obese	−3.9*** (0.4)	−6.8*** (0.3)	2.9*** (0.4)
Low education (<14 years)			
Nonsmokers, not obese	23.9 (0.2)	15.7 (0.2)	8.2 (0.2)
Smokers, not obese	21.4 (0.2)	13.7 (0.2)	7.7 (0.2)
Nonsmokers, obese	22.5 (0.3)	10.7 (0.3)	11.7 (0.4)
Smokers, obese	20.2 (0.4)	9.1 (0.3)	11.1 (0.4)
Loss(−)/gain(+)^a^			
Smoker, not obese	−2.5*** (0.3)	−2.0*** (0.3)	−0.5 (0.3)
Nonsmoker obese	−1.4*** (0.4)	−4.9*** (0.3)	3.5*** (0.4)
Smoker, obese	−3.7*** (0.4)	−6.6*** (0.3)	2.9*** (0.4)

*Notes*: ^a^Relative to nonsmoker, not obese.

*p < .05. **p < .01. ***p < .001.

**Table 3.** Total Life Expectancy, Years Free of Activities of Daily Living (ADL) Disability, and Years With ADL Disability (*SE*s in parentheses) at Age 65, by Risk Factor, Education and Gender

	Total Life Years (*SE*)	Years Free of ADL Disability (*SE*)	Years With ADL Disability (*SE*)
Men			
High education (≥14 years)			
Nonsmokers, not obese	20.9 (0.4)	17.3 (0.5)	3.6 (0.3)
Smokers, not obese	18.2 (0.3)	15.3 (0.3)	2.9 (0.2)
Nonsmokers, obese	19.5 (0.7)	15.7 (0.8)	3.7 (0.5)
Smokers, obese	17.3 (0.6)	14.2 (0.6)	3.2 (0.4)
Loss(−)/gain(+)^a^			
Smoker, not obese	−2.6*** (0.6)	−2.0*** (0.6)	−0.7 (0.4)
Nonsmoker obese	−1.4 (0.8)	−1.6 (0.9)	0.2 (0.6)
Smoker, obese	−3.5*** (0.8)	−3.1*** (0.8)	−0.4 (0.5)
Low education (<14 years)			
Nonsmokers, not obese	19.7 (0.5)	15.8 (0.5)	3.9 (0.4)
Smokers, not obese	17.2 (0.4)	14.0 (0.4)	3.2 (0.3)
Nonsmokers, obese	18.4 (0.7)	14.2 (0.8)	4.1 (0.5)
Smokers, obese	16.3 (0.6)	12.8 (0.6)	3.5 (0.4)
Loss(−)/gain(+)^a^			
Smoker, not obese	−2.5*** (0.6)	−1.8** (0.7)	−0.7 (0.4)
Nonsmoker obese	−1.4 (0.9)	−1.6 (1.0)	0.2 (0.6)
Smoker, obese	−3.4*** (0.8)	−3.0*** (0.8)	−0.4 (0.6)
Women			
High education (≥14 years)			
Nonsmokers, not obese	24.8 (0.2)	20.6 (0.2)	4.2 (0.2)
Smokers, not obese	22.3 (0.2)	18.6 (0.2)	3.7 (0.2)
Nonsmokers, obese	23.3 (0.3)	17.4 (0.3)	5.8 (0.3)
Smokers, obese	20.9 (0.3)	15.7 (0.3)	5.2 (0.3)
Loss(−)/gain(+)^a^			
Smoker, not obese	−2.5*** (0.3)	−2.0*** (0.3)	−0.5* (0.2)
Nonsmoker obese	−1.5*** (0.4)	−3.2*** (0.3)	1.6*** (0.3)
Smoker, obese	−3.9*** (0.4)	−4.9*** (0.3)	1.0** (0.3)
Low education (<14 years)			
Nonsmokers, not obese	24.0 (0.2)	19.7 (0.2)	4.3 (0.2)
Smokers, not obese	21.6 (0.2)	17.8 (0.2)	3.8 (0.2)
Nonsmokers, obese	22.4 (0.3)	16.4 (0.3)	5.9 (0.3)
Smokers, obese	20.1 (0.3)	14.8 (0.3)	5.3 (0.3)
Loss(−)/gain(+)^a^			
Smoker, not obese	−2.4*** (0.3)	−1.9*** (0.3)	−0.5* (0.2)
Nonsmoker obese	−1.7*** (0.4)	−3.3*** (0.3)	1.6*** (0.3)
Smoker, obese	−4.0*** (0.4)	−4.9*** (0.3)	1.0** (0.3)

*Notes*: ^a^Relative to nonsmoker, not obese.

*p < .05. **p < .01. ***p < .001.

**Figure 1.**
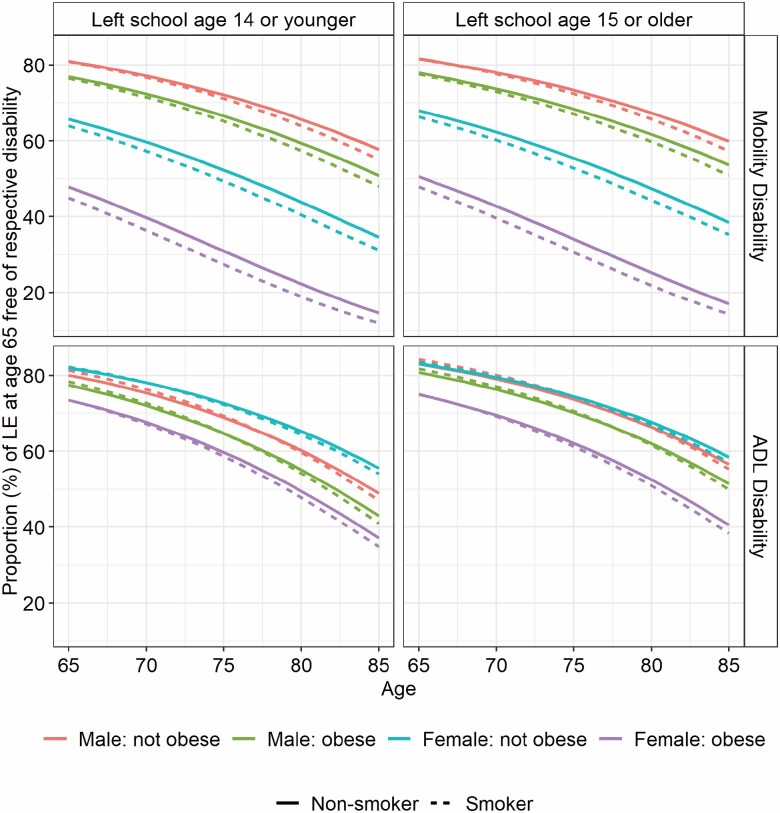
Proportion of remaining life at different ages spent with mobility disability, and ADL disability, by sex and groups defined by smoking and/or obesity.

Men and women aged 65 who smoked but were not obese could expect to live 2.5 years less than nonobese nonsmokers (*p* < .001), around 2.0 fewer years free of mobility disability (*p* < .001) and around half a year less with mobility disability (men: 0.5 years, *p* = .20; women: 0.4 years, *p* = .11) (Table 2). The stronger effect of obesity on transitions for women was reflected in LE, but, in contrast to smoking, the effect was greater for years free of mobility disability than total life years, resulting in large differences in the proportion of remaining life free of mobility disability (Figure 1). Indeed, at age 65, obese nonsmoking women lived on average 1.6 years less (*p* < .001) than their nonobese, nonsmoking counterparts but had 5.1 years fewer free of mobility disability (*p* < .001) and 3.5 years more with mobility disability (*p* < .001). These differential effects of obesity and smoking on total life years and years free of mobility disability between men and women were still evident for those initially free of disability from status-based life tables (Supplementary Table 3).

The effect of smoking on ADL disability was very similar to that on mobility disability. However, obesity alone resulted in smaller reductions in years free of ADL disability, smaller gains in years with ADL disability (Table 3), and smaller differences in the proportion of remaining life free of ADL disability (Figure 1). Nevertheless women aged 65 who smoked and were obese could expect 3.9 fewer years of life (*p* < .001), 4.9 fewer years free of ADL disability (*p* < .001), and 1.0 more years with ADL disability (*p* = .003) compared to their nonobese nonsmoking counterparts (Table 3).

### Sensitivity Analysis

After exclusion of the ALSWH, we recalculated the effect of risk factors on transition probabilities (Supplementary Table2) and years with and without mobility disability at age 65 (Supplementary Table 4) and ADL disability (Supplementary Table 5). The relative probabilities of transition were generally similar although confidence intervals were wider due to fewer observed transitions. Without ALSWH, obesity still increased the likelihood of incident mobility disability (but no longer ADL disability) and reduced the likelihood of recovery from ADL disability (but not longer mobility disability) (Supplementary Table 2). Patterns between the risk factor groups for LE and DFLE were unchanged, although increases in years with disability for women who were obese nonsmokers (compared to nonobese nonsmokers) were attenuated (Supplementary Tables 4 and 5).

## Discussion

Global burden of disease studies have shown large gains in LE, but often with increased years lived with disability (35) and risk factors such as smoking and obesity are major contributors (36). Ours is the first study to examine the impact of smoking and obesity on DFLE using 2 measures of disability reflecting different stages in the disablement process. Men and women who smoked but were not obese lived around 2.5 years less and 2 years fewer free of disability, irrespective of the disability measure, with therefore around half a year less with disability. Thus, elimination of smoking would result in an increase in total life years but not all the years would be free of disability. In contrast, obesity had a more marked effect in women and for mobility disability compared to ADL disability, with reductions of 1.6 years fewer overall but 5.1 years fewer free of mobility disability and an extra 3.5 years with mobility disability at age 65. Elimination of obesity therefore would result in smaller gains in total life years but an absolute reduction in years with disability.

The Global Burden of Disease study identified obesity as a key driver of disease burden in middle- and high-income countries, and with changing lifestyles, obesity-related diseases are also emerging in low-middle income countries, concomitant with population aging (37). This increase in obesity is likely to produce increases in years of life in poor health, particularly since some studies have shown that older obese adults may have longer LE, termed the “obesity paradox” (38). In Canada, obesity was associated with loss of health-adjusted LE, with little impact on total LE. Moreover, while overweight was associated with increases in total life years, there was a reduction in healthy life for people in this category (39). We compared obese versus nonobese categories (nonobese including normal BMI and overweight), and therefore the magnitude of obesity effects on LE and DFLE in our study are likely to be more conservative than if we had compared obese and normal BMI categories.

Much of the previous literature on the effect of obesity on life and health expectancy has emanated from the United States. Projections for increases in healthy LE, based on Sullivan’s method, highlight the negative impact of obesity, smoking, and socioeconomic disadvantage (40). The Behavioral Risk Factor Surveillance System found smokers had shorter LE and lower quality of life scores, and therefore substantially fewer quality-adjusted years, while obesity was strongly associated with lower quality of life scores, but did not have a strong effect on LE (41). Similarly, for both men and women in the Health and Retirement Survey, obesity was associated with a small reduction in total LE at age 50, but a large reduction in DFLE (2.3 years for men and 4.8 years for women). In contrast, smokers had shorter total LE, but lived fewer years with disability when compared to nonsmokers (42). A European study across 4 countries in Europe (England, Finland, France, and Sweden) found that men and women with multiple (at least 2) risk factors of smoking, obesity, or physical inactivity could expect to live on average 8 years less in good health between ages 50 and 75, when compared to those with no risk factors (43).

These effects have particular importance considering the 2 widespread trends of population aging and rising rates of obesity, while smoking rates have been decreasing, and the effects of smoking on both LE and disability may be less in future generations. Increasing rates of obesity are associated with large increase in diseases, including diabetes and arthritis, which are in turn associated with a heavy burden of disability, particularly mobility limitations (44). There may be a more direct effect of obesity on mobility limitations through inflammatory processes, as well as through a reduced ratio of fat-free mass to total body mass (45). Additionally, there may be a bidirectional effect with disability causing obesity, although similar obesity effects were evident in those initially free of mobility disability from status-based tables.

In our study, the effects of obesity were greatest for mobility disability, and for women. When we removed the largest, all-women, study there was some attenuation of the effect of obesity on disability but significant differences in LE and DFLE between the risk factor groups remained, suggesting that the effects in women were not simply due to the greater sample size. Other studies have also noted greater effects of obesity on quality of life for women (46,47), but the mechanisms are not clear. It is possible that the ratio of lean body mass to adipose tissue moderates the impact of obesity in women since obese males have higher percentage lean body mass that may protect them from physical disability (48). Other factors may include different end-organ effects of the inflammatory and hormonal changes associated with obesity. However, there is also a possibility that the effects are mediated by comorbidities, or psychosocial factors, including attitudes to women’s weight and shape (46). In addition, there is evidence that the negative effect of obesity on disability and mortality in older people may have changed over time, with greater reductions in the proportion of life spent with ADL disability for the obese and women (49).

Limitations of our study lie in the nature of the data (intervals between follow-up and age of studies) and in the measures included (disability, obesity) and not included (physical activity, percentage lean body mass, confounding factors). In our study, the intervals between follow-ups range from 1 year (ALSWH) to 5 years (BMES) and longer follow-up intervals could miss transitions from disability-free to disability. The largest study, ALSWH, had 1-year follow-ups over the first 3 years; we re-ran analyses excluding this study and conclusions were unchanged, suggesting longer intervals may not have a large effect in our study. The studies we included mostly started in the 1990s and since then there have been considerable changes in health care and health literacy, not only in Australia. Education norms have changed considerably for Australian women in younger cohorts, although again we found little difference in the impact of obesity on disability between the education groups. Smoking rates have declined in Australia and are continuing to do so, although mid-life obesity is increasing. Thus, our results of more years with disability being associated with obesity, particularly for women, suggest that, as smokers die early, contemporary cohorts may have more years with disability. This hypothesis is not supported however by recent data from the U.S. Health and Retirement Study which shows reductions in the proportion of life spent with ADL disability for older Americans aged 70 years and older across 2 periods, 1993–1998 and 2010–2014 (49). Moreover, there was strong evidence that improvements in active LE, greatest among the obese and women, were likely to be due to changes in the obesity–disability–mortality relationship than to changes in the BMI composition of the older population. However, their measure of disability based on inability to perform ADLs differed from ours, and we found the strongest impact of obesity to be on mobility disability, which manifests earlier in the disablement process. Nevertheless, older datasets provide useful baseline reference for future studies. Harmonization across the studies meant that disability items that could be considered equivalent were limited. Our measure of mobility, walking 1 km, did not specify whether this was with or without aids. Although it may be that the highly educated group may have been more able to purchase mobility aids when they first had problems than people with lower levels of education (and thus explaining the earlier incidence of mobility disability in the less educated group), the effect of obesity on years with mobility disability were very similar across the education groups. We considered obese to be a BMI of 30 or over, since there were insufficient numbers to look at a more nuanced measure of obesity that differentiates Class I obesity (BMI = 30.0–34.9) from Class II (BMI = 35.0–39.9) or Class III (BMI ≥ 40.0). This is especially relevant given evidence suggesting that Class I obesity may be protective for some chronic conditions (38). Indeed, further subdivision by high or low percentage lean mass may also have been informative as high lean mass with obesity may partly explain the obesity paradox (38). Given the small numbers of participants who were underweight, and the high risk of mortality in this group, we excluded underweight participants. However, as BMI was only measured at baseline, participants may have become obese or underweight over the period of follow-up. In terms of the relationship between obesity and mortality, a recent study suggests that the weaker relationship in older than younger people may be confounded by historical changes in weight and adiposity rather than changes in body composition with aging (50). For the obesity–disability relationship, the effect of participants becoming obese after baseline would be to dilute the impact of obesity on disability, while the effect of obese persons at baseline losing weight would be most likely to increase the impact of obesity on recovery. Thus, our results are likely to be more conservative than if we had included obesity status closer to the outcome. Finally, we only considered education as a potential confounding factor because of stratified sample size limitations.

The strengths of our study were the large sample of over 20 000 adults participating in 5 longitudinal studies across Australia. All but one study had multiple follow-ups from baseline, thereby providing enough transitions between no disability, disability, and death to estimate incidence, recovery, and state-specific death rates. In addition, previous harmonization of the disability items meant we could explore effects across the spectrum of disability severity. Modeling the effect of risk factors on mortality alongside disability, in DFLE, rather than simply on the incidence of disability is important, particularly when risk factors such as smoking and obesity have differential effects on mortality and disability.

Our findings emphasize the need to invest in prevention programs to reduce rates of smoking and obesity. At a population level, the effects of obesity on disability are considerable, and obese people are living as long as healthy weight counterparts but with more years with disability. These impacts are greater on mobility limitations, than for ADL. This effect may become a “vicious cycle” whereby obese individuals may find it harder to exercise, and thereby experience further losses in functional capacity and limits to participation. While efforts to prevent obesity, starting in childhood, are an obvious imperative, there may be benefit in interventions with obese adults to increase fitness and lean body mass, to maintain mobility and reduce the impact of arthritis and other causes of physical limitation. Future studies should seek to include historical changes in weight and a wider spectrum of disability severity to better quantify the extent to which how the increasing prevalence of obesity reduces quality of life at older ages.

## Funding

DYNOPTA was funded by the National Health and Medical Research Council (NHMRC: grant no. 410215); ALSWH is funded by the Australian Department of Health. This work was partially supported by a travel grant from the Australian Centre of Excellence in Population Ageing Research (CEPAR) CE170100005 to C.J. and a personal fellowship awarded to A.K. from Newcastle University, UK. The views expressed are those of the author(s) and not necessarily those of Newcastle University. K.A is funded by NHMRC Fellowship 1102694.

## Supplementary Material

glaa290_suppl_Supplementary_Materials
