# Supplementary material for: The Impact of Smoking and Obesity on Disability-Free Life Expectancy in Older Australians
Source: J Gerontol A Biol Sci Med Sci. 2020 Nov 29;76(7):1265–72. doi: 10.1093/gerona/glaa290 (PMC8202145; doi:10.1093/gerona/glaa290)
Supplement: glaa290_suppl_Supplementary_Materials [file glaa290_suppl_supplementary_materials.docx]

**Supplementary material for: Kingston A, Byles J, Kiely K, Anstey K, Jagger C. The impact of smoking and obesity on disability-free life expectancy in older Australians**

**eMethods**

The studies used in this analysis came from the Dynamic Analyses to Optimise Ageing (DYNOPTA) project in Australia, pooled data from Australian longitudinal studies of ageing. We selected studies that had information on both measures of disability (as well as obesity and smoking), these being:

1. The Australian Longitudinal Study of Ageing (ALSA) – began in 1992 with participants from Adelaide, South Australia aged 65 years or over, living in the community or residential care, and with follow-ups at 1,2,3,5 and 8 years
2. The Australian Longitudinal Study of Women’s Health (ALSWH) – a national sample of women in three birth cohorts: 1973-78, 1946-51, and 1921-26. We use the 1921-26 cohort who were aged 70-75 when first interviewed in 1996 and who were followed up in 1999, 2002, 2005 and 2008
3. The Blue Mountains Eye Study (BMES) – a semi-urban population sample from the Blue Mountains area of New South Wales, first interviewed in 1992 aged 49-97 years with two follow-up interviews in 1997 and 2002.
4. The Melbourne Longitudinal Studies on Healthy Ageing (MELSHA) – a non-institutionalised population sample from Melbourne, Victoria, first interviewed in 1994 aged 65 years and over with 5 follow-up interviews at approximately two year intervals
5. The Personality and Health Through Life (PATH) – a random sample of older people from Canberra and Queanbeyan, New South Wales, first interviewed in 2001 aged 60-64 years with one follow-up interview in 2005

**eTable 1: Observed transitions from no disability to disability and to death, by sex and type of disability**

|  |  |  |  | To |  |
| --- | --- | --- | --- | --- | --- |
|  |  | From | No disability | Disability | Death |
| Mobility disability | Men | No disability | 4547 | 561 | 746 |
|  |  | Disability | 291 | 614 | 511 |
|  | Women | No disability | 17508 | 3982 | 1577 |
|  |  | Disability | 1976 | 5626 | 1801 |
| ADL disability | Men | No disability | 3133 | 388 | 516 |
|  |  | Disability | 120 | 125 | 207 |
|  | Women | No disability | 22230 | 2128 | 2118 |
|  |  | Disability | 1165 | 1285 | 862 |

**eTable 2: Relative probabilities of transitions (and 95% confidence intervals in parentheses) for the main effects of low education, smoking, and obesity, by sex and type of disability**

|  |  | Men | Women  (All) | Women  (excluding ALSWH) |
| --- | --- | --- | --- | --- |
| **Mobility disability** |  |  |  |  |
| Incident disability | Low education | *1.25 (1.04, 1.51)* | *1.14 (1.07, 1.22)* | *1.21 (1.02, 1.43)* |
|  | Smoker | 1.10 (0.91, 1.34) | *1.09 (1.02, 1.16)* | 0.98 (0.82, 1.17) |
|  | Obese | 1.18 (0.92, 1.52) | *1.88 (1.72, 2.05)* | *1.96 (1.61, 2.38)* |
| Recovery from disability | Low education | 1.21 (0.94, 1.58) | 1.01 (0.92, 1.11) | 1.11 (0.89, 1.40) |
|  | Smoker | 0.97 (0.73, 1.28) | *0.78 (0.70, 0.86)* | 0.82 (0.65, 1.03) |
|  | Obese | *0.69 (0.49, 0.98)* | *0.70 (0.62, 0.80)* | 0.94 (0.73, 1.20) |
| No disability to death | Low education | 1.11 (0.86, 1.44) | *1.25 (1.03, 1.52)* | *1.50 (1.08, 2.09)* |
|  | Smoker | *1.73 (1.28, 2.34)* | *1.64 (1.35, 1.99)* | *1.91 (1.36, 2.66)* |
|  | Obese | 0.83 (0.55, 1.27) | 1.15 (0.84, 1.58) | 1.09 (0.68, 1.75) |
| Disability to death | Low education | 1.09 (0.90, 1.31) | 0.99 (0.91, 1.09) | 0.95 (0.79, 1.14) |
|  | Smoker | 1.09 (0.89, 1.33) | *1.25 (1.14, 1.37)* | 1.19 (0.97, 1.45) |
|  | Obese | 1.16 (0.91, 1.49) | 0.91 (0.81, 1.02) | 1.10 (0.90, 1.35) |
|  |  |  |  |  |
| **ADL disability** |  |  |  |  |
| Incident disability | Low education | *1.31 (1.03, 1.67)* | *1.22 (1.12, 1.34)* | 0.95 (0.75, 1.20) |
|  | Smoker | 0.93 (0.71, 1.21) | *1.14 (1.04, 1.25)* | *0.75 (0.59, 0.96)* |
|  | Obese | 1.18 (0.86, 1.63) | *1.77 (1.59, 1.98)* | 1.24 (0.96, 1.60) |
| Recovery from disability | Low education | 1.01 (0.64, 1.57) | 1.02 (0.90, 1.16) | 0.76 (0.51, 1.14) |
|  | Smoker | 0.85 (0.54, 1.34) | 0.88 (0.77, 1.00) | *0.59 (0.40, 0.88)* |
|  | Obese | 0.76 (0.44, 1.31) | *0.80 (0.68, 0.93)* | *0.56 (0.36, 0.86)* |
| No disability to death | Low education | 1.16 (0.90, 1.49) | 0.99 (0.87, 1.13) | 1.16 (0.89, 1.51) |
|  | Smoker | *2.09 (1.46, 2.98)* | *1.49 (1.31, 1.69)* | *1.61 (1.24, 2.09)* |
|  | Obese | 1.00 (0.69, 1.45) | 1.04 (0.85, 1.27) | 1.18 (0.85, 1.62) |
| Disability to death | Low education | 0.98 (0.79, 1.21) | *1.13 (1.00, 1.29)* | 1.11 (0.89, 1.38) |
|  | Smoker | 0.98 (0.78, 1.22) | *1.25 (1.10, 1.43)* | *1.30 (1.01, 1.67)* |
|  | Obese | 1.13 (0.84, 1.50) | 1.00 (0.86, 1.16) | 1.24 (0.96, 1.59) |

Note. ALSWH - Australian Longitudinal Study of Women’s Health

**eTable 3: Status-based total life expectancy, years free of mobility disability, and years with mobility disability (standard errors in parentheses) at age 65 for those initially free of mobility disability, by risk factor, education and gender**

|  | Total life years  (SE) | Years free of mobility disability (SE) | Years with mobility disability (SE) |
| --- | --- | --- | --- |
| MEN |  |  |  |
| **High education (≥14 years)** |  |  |  |
| Non-smokers, not obese | 21.0 (0.4) | 17.3 (0.4) | 3.7 (0.3) |
| Smokers, not obese | 18.4 (0.3) | 15.2 (0.3) | 3.2 (0.2) |
| Non-smokers, obese | 19.9 (0.7) | 15.7 (0.7) | 4.1 (0.5) |
| Smokers, obese | 17.8 (0.6) | 14.1 (0.6) | 3.7 (0.4) |
| *Loss(-)/gain(+)^a^* |  |  |  |
| *Smoker, not obese* | -2.5*** (0.6) | -2.1*** (0.5) | -0.5 (0.3) |
| *Non-smoker obese* | -1.1 (0.8) | -1.5 (0.8) | 0.4 (0.5) |
| *Smoker, obese* | -3.2*** (0.7) | -3.2*** (0.7) | 0.0 (0.5) |
| **Low education (<14 years)** |  |  |  |
| Non-smokers, not obese | 20.0 (0.5) | 16.4 (0.5) | 3.7 (0.3) |
| Smokers, not obese | 17.6 (0.4) | 14.4 (0.3) | 3.2 (0.2) |
| Non-smokers, obese | 18.8 (0.7) | 14.7 (0.7) | 4.1 (0.4) |
| Smokers, obese | 16.9 (0.6) | 13.2 (0.6) | 3.7 (0.4) |
| *Loss(-)/gain(+)^a^* |  |  |  |
| *Smoker, not obese* | -2.5*** (0.6) | -2.0** (0.6) | -0.5 (0.4) |
| *Non-smoker obese* | -1.2 (0.8) | -1.6 (0.9) | 0.4 (0.5) |
| *Smoker, obese* | -3.2*** (0.7) | -3.2*** (0.7) | 0.0 (0.5) |
| WOMEN |  |  |  |
| **High education (≥14 years)** |  |  |  |
| Non-smokers, not obese | 24.6 (0.2) | 17 (0.2) | 7.6 (0.2) |
| Smokers, not obese | 22.2 (0.2) | 15.1 (0.2) | 7.1 (0.2) |
| Non-smokers, obese | 23.0 (0.3) | 12.3 (0.2) | 10.7 (0.3) |
| Smokers, obese | 20.8 (0.3) | 10.9 (0.2) | 10.0 (0.3) |
| *Loss(-)/gain(+)^a^* |  |  |  |
| *Smoker, not obese* | -2.4*** (0.3) | -1.9*** (0.2) | -0.6* (0.3) |
| *Non-smoker obese* | -1.6*** (0.4) | -4.7*** (0.3) | 3.1*** (0.4) |
| *Smoker, obese* | -3.8*** (0.4) | -6.1*** (0.3) | 2.3*** (0.4) |
| **Low education (<14 years)** |  |  |  |
| Non-smokers, not obese | 23.9 (0.2) | 16.0 (0.2) | 7.9 (0.2) |
| Smokers, not obese | 21.4 (0.2) | 14.1 (0.2) | 7.3 (0.2) |
| Non-smokers, obese | 22.6 (0.3) | 11.5 (0.2) | 11.1 (0.3) |
| Smokers, obese | 20.3 (0.4) | 10.1 (0.2) | 10.2 (0.4) |
| *Loss(-)/gain(+)^a^* |  |  |  |
| *Smoker, not obese* | -2.5*** (0.3) | -1.9*** (0.3) | -0.6* (0.3) |
| *Non-smoker obese* | -1.4** (0.4) | -4.5*** (0.3) | 3.2*** (0.4) |
| *Smoker, obese* | -3.6*** (0.4) | -5.9*** (0.3) | 2.3*** (0.4) |

*p<0.05; **p<0.01, ***p<0.001 ^a^relative to non-smoker, not obese

**eTable 4: Total life expectancy, years free of mobility disability, and years with mobility disability (standard errors in parentheses) at age 65, by risk factor and education, women excluding the Australian Longitudinal Study of Women’s Health**

|  | Total life years  (SE) | Years free of mobility disability (SE) | Years with mobility disability (SE) |
| --- | --- | --- | --- |
| WOMEN |  |  |  |
| **High education (≥14 years)** |  |  |  |
| Non-smokers, not obese | 24.2 (0.4) | 18.0 (0.4) | 6.1 (0.3) |
| Smokers, not obese | 21.5 (0.4) | 16.2 (0.4) | 5.4 (0.3) |
| Non-smokers, obese | 21.6 (0.5) | 13.6 (0.5) | 8.0 (0.5) |
| Smokers, obese | 19.4 (0.6) | 12.2 (0.6) | 7.2 (0.5) |
| *Loss(-)/gain(+)^a^* |  |  |  |
| *Smoker, not obese* | -2.6*** (0.6) | -1.9** (0.6) | -0.8 (0.5) |
| *Non-smoker obese* | -2.6*** (0.7) | -4.4*** (0.7) | 1.8** (0.6) |
| *Smoker, obese* | -4.8*** (0.7) | -5.8*** (0.7) | 1.0 (0.6) |
| **Low education (<14 years)** |  |  |  |
| Non-smokers, not obese | 23.1 (0.4) | 16.8 (0.4) | 6.3 (0.3) |
| Smokers, not obese | 20.1 (0.5) | 14.7 (0.5) | 5.4 (0.4) |
| Non-smokers, obese | 20.9 (0.5) | 12.7 (0.5) | 8.2 (0.5) |
| Smokers, obese | 18.4 (0.7) | 11.2 (0.6) | 7.3 (0.6) |
| *Loss(-)/gain(+)^a^* |  |  |  |
| *Smoker, not obese* | -3.0*** (0.7) | -2.1** (0.6) | -0.9 (0.5) |
| *Non-smoker obese* | -2.2** (0.7) | -4.1*** (0.6) | 1.9** (0.6) |
| *Smoker, obese* | -4.7*** (0.8) | -5.7*** (0.7) | 1.0 (0.7) |

*p<0.05; **p<0.01, ***p<0.001 ^a^relative to non-smoker, not obese

**eTable 5: Total life expectancy, years free of ADL disability, and years with ADL disability (standard errors in parentheses) at age 65, by risk factor and education, women excluding the Australian Longitudinal Study of Women’s Health**

|  | Total life years  (SE) | Years free of ADL disability (SE) | Years with ADL disability (SE) |
| --- | --- | --- | --- |
| WOMEN |  |  |  |
| **High education (≥14 years)** |  |  |  |
| Non-smokers, not obese | 24.0 (0.4) | 18.6 (0.4) | 5.4 (0.4) |
| Smokers, not obese | 21.3 (0.4) | 17.1 (0.5) | 4.2 (0.4) |
| Non-smokers, obese | 21.7 (0.5) | 15.5 (0.6) | 6.3 (0.6) |
| Smokers, obese | 19.4 (0.6) | 14.5 (0.6) | 4.9 (0.6) |
| *Loss(-)/gain(+)^a^* |  |  |  |
| *Smoker, not obese* | -2.7*** (0.7) | -1.5* (0.6) | -1.2* (0.5) |
| *Non-smoker obese* | -2.3** (0.7) | -3.2** (0.8) | 0.9 (0.7) |
| *Smoker, obese* | -4.6*** (0.7) | -4.2** (0.8) | -0.5 (0.7) |
| **Low education (<14 years)** |  |  |  |
| Non-smokers, not obese | 22.9 (0.4) | 17.7 (0.4) | 5.3 (0.4) |
| Smokers, not obese | 20.3 (0.5) | 16.3 (0.6) | 4.0 (0.4) |
| Non-smokers, obese | 20.7 (0.5) | 14.7 (0.7) | 6.1 (0.6) |
| Smokers, obese | 18.4 (0.6) | 13.8 (0.7) | 4.7 (0.6) |
| *Loss(-)/gain(+)^a^* |  |  |  |
| *Smoker, not obese* | -2.6*** (0.7) | -1.4* (0.7) | -1.2* (0.6) |
| *Non-smoker obese* | -2.2** (0.7) | -3.0*** (0.8) | 0.8 (0.7) |
| *Smoker, obese* | -4.5*** (0.7) | -3.9*** (0.8) | -0.6 (0.7) |

*p<0.05; **p<0.01, ***p<0.001 ^a^relative to non-smoker, not obese
